# ER Alpha Rapid Signaling Is Required for Estrogen Induced Proliferation and Migration of Vascular Endothelial Cells

**DOI:** 10.1371/journal.pone.0152807

**Published:** 2016-04-01

**Authors:** Qing Lu, Gavin R. Schnitzler, Kazutaka Ueda, Lakshmanan K. Iyer, Olga I. Diomede, Tiffany Andrade, Richard H. Karas

**Affiliations:** Molecular Cardiology Research Institute, Tufts Medical Center, Boston, Massachusetts, United States of America; II Università di Napoli, ITALY

## Abstract

Estrogen promotes the proliferation and migration of vascular endothelial cells (ECs), which likely underlies its ability to accelerate re-endothelialization and reduce adverse remodeling after vascular injury. In previous studies, we have shown that the protective effects of E2 (the active endogenous form of estrogen) in vascular injury require the estrogen receptor alpha (ERα). ERα transduces the effects of estrogen via a classical DNA binding, “genomic” signaling pathway and via a more recently-described “rapid” signaling pathway that is mediated by a subset of ERα localized to the cell membrane. However, which of these pathways mediates the effects of estrogen on endothelial cells is poorly understood. Here we identify a triple point mutant version of ERα (KRR ERα) that is specifically defective in rapid signaling, but is competent to regulate transcription through the “genomic” pathway. We find that in ECs expressing wild type ERα, E2 regulates many genes involved in cell migration and proliferation, promotes EC migration and proliferation, and also blocks the adhesion of monocytes to ECs. ECs expressing KRR mutant ERα, however, lack all of these responses. These observations establish KRR ERα as a novel tool that could greatly facilitate future studies into the vascular and non-vascular functions of ERα rapid signaling. Further, they support that rapid signaling through ERα is essential for many of the transcriptional and physiological responses of ECs to E2, and that ERα rapid signaling in ECs, in vivo, may be critical for the vasculoprotective and anti-inflammatory effects of estrogen.

## Introduction

Cardiovascular disease is the leading cause of death, for both men and women, in the developed world. Women, however, have a much lower incidence of cardiovascular disease than men until they reach menopause, suggesting an important role for endogenous estrogen. Indeed, as described further below, studies in animal models strongly support that estrogen has vasculoprotective functions. Unfortunately, clinical studies indicate that the vascular effects of estrogen are complex, with the vasculoprotective effects observed in younger women being lost in women over age 60 [1–6]. Furthermore, estrogen treatments are associated with significant detrimental effects in other tissues–including feminization in men, and increased risks of breast cancer, uterine cancer and thrombosis in women [6–9]. These complications indicate that before we can translate the potential vascular protective effects of estrogen into therapies to prevent or treat cardiovascular disease, we must have a much better understanding of the mechanisms by which estrogen protects against vascular injury and disease.

Mouse knock out studies in our research group have shown that the estrogen receptor alpha transcription factor (ERα) is required for the ability of 17β estradiol (E2, the active, natural form of estrogen) to protect against pathologic vascular injury responses, including inhibition of injury-associated increases in smooth muscle cell (SMC) growth, vascular medial area and fibrosis [10]. In addition, other studies have shown that ERα is required for E2-dependent protection from atherosclerosis (including inhibition of plaque formation and complexity, and reduction of circulating cholesterol [11], for review see [6]), and for the ability of E2 to promote re-endothelialization after vascular injury [12]. By contrast, the second ER homologue, ERβ is dispensable for the protective effects of estrogen in vascular injury [12–14]. It has also been shown that E2 reduces the production of inflammatory cytokines and neutrophil chemotaxis in injured vessels, although whether this affects long term injury outcomes or requires ERα is not known [15, 16]. At a cellular level, we and others have shown that E2 reduces the proliferation of vascular SMCs, whose growth in response to injury represents a central pathophysiologic component of adverse vascular remodeling [17–20]. E2 also promotes the proliferation and migration of vascular endothelial cells (ECs), an essential aspect of vascular repair after injury [20, 21].

ERα is a transcription factor (TF) that, when bound by E2, moves to the nucleus, binds to specific sites on chromatin, and activates or represses target gene transcription (the “classical genomic” pathway). In addition to its chromatin binding functions, a fraction of cellular ERα is palmitoylated, and forms signaling complexes in caveolae on the plasma membrane [22–24]. This membrane-bound ERα, through interaction with specific adaptor proteins, activates several important cellular kinases, including c-Src, PI3-kinase, Akt and ERK1/2. For simplicity, we will refer to this kinase activation pathway, here, as the “rapid” pathway. One of the best characterized functions of this rapid pathway is to activate endothelial nitric oxide synthase (eNOS), resulting in vasorelaxation within minutes of the addition of E2 [25, 26]. However, until recently, it was unclear whether rapid signaling was relevant to longer term responses to vascular insult or injury.

We recently demonstrated that rapid signaling is mediated though the binding of ERα or ERβ to the adaptor molecule striatin, such that loss of striatin or disruption of ER-striatin interactions eliminates the rapid signaling effects of E2, without altering its genomic effects [27]. We developed a novel transgenic mouse model which expresses a peptide that blocks the interactions between ERα and ERβ and striatin, and is therefore deficient in rapid signaling responses to E2 (the disrupting peptide, or “DPM”, mouse, [20]). Strikingly, the ability of E2 to protect against SMC proliferation and vascular remodeling after carotid artery wire injury, that is seen in WT mice, was lost in DPM mice, similar to what was seen in ERα knockout mice. Consistent with this in vivo phenotype, cell culture studies using the disrupting peptide showed that rapid signaling is required for the ability of E2 to inhibit primary SMC proliferation, and to promote the migration and proliferation of EC cell lines [19, 20]. Notably, in expression microarray analyses, we found that genes regulated by E2 in DPM mouse aortas differ greatly from those regulated by E2 in WT aortas, indicating that rapid signaling plays an important role in vascular transcriptional regulatory responses to E2. These observations have challenged the dominant paradigm that gene regulation and long term vascular physiological effects of E2 will be almost entirely mediated by the classic genomic pathway requiring ER binding to chromatin ([20], and accompanying editorial review [28], and [29]).

The disrupting peptide blocks the interaction of striatin with ERα as well as ERβ. It also inhibits the association of PP2A with striatin [19, 30], and could potentially block the interaction of other proteins that bind to the same domain on striatin. Thus, while the similarity in phenotypes between ERαKO and DPM mice suggests a requirement for rapid signaling through ERα in mediating the protective effects of E2, the DPM model is not precise enough to allow the unambiguous identification of E2/ERα-specific rapid signaling functions, or the dissection of the mechanisms underlying these functions. Here, we describe the identification of a “KRR” triple point mutation of ERα that is deficient in rapid signaling through striatin but fully active for genomic signaling. We use this KRR mutant ERα to show that rapid signaling through ERα is critical for gene regulation by E2/ERα in ECs, and for the ability of E2 to inhibit monocyte adhesion to ECs and to promote EC proliferation and migration. These observations suggest that rapid signaling, specifically through ERα, will be essential for relevant responses of vascular cells to E2, and establish KRR mutant ERα as a novel model to facilitate the precise identification of the vascular cell physiological functions and molecular mechanisms of rapid signaling through ERα.

## Materials and Methods

### Plasmids

The WT ERα plasmid was constructed by cloning the full-length WT human ERα into pCDNA3.1 vector, as described in [27, 31]. The plasmid for GST pull-down experiments was constructed by cloning the PCR-derived ERα 176–253 fragment into pGEX-4T-1 (Amersham Pharmacia). ERα full-length mutants and GST ERα 176–253 mutants were constructed using site-directed mutagenesis (Stratagene, La Jolla, CA). The presence of the correct sequence in all vector inserts was confirmed by DNA sequencing. The eNOS expression plasmid was a kind gift from Dr. M. Mendelsohn.

### Cell lines and culture

COS1 cells were obtained from ATCC (American Type Culture Collection) and were grown in DMEM with 10% fetal bovine serum. EAhy926 cells [32] (a human umbilical vein endothelial cell hybrid, kind gift of C.J. Edgell, University of North Carolina at Chapel Hill), that do not express ERα or ERβ, were transfected with either the WT or KRR mutant ERα expression plasmid ([31], “WT_hEC” and “KRR_hEC” cells) or with control backbone vector pCDNA 3.1 (Invitrogen, “Ctrl_hEC” cells) using PolyFect transfection reagent (Qiagen). After 24 hours the cells were placed in selective media with 5 μg/ml puromycin (Sigma) for 2 to 3 weeks. Eight to ten single colonies were selected and maintained in the presence of 2 μg/ml puromycin. For RNA isolation, EAhy926 stable cells were grown in 6-well plates to 80% confluence, switched to serum free medium for 24 hours, and then treated with 10 nM E2 or ethanol vehicle for 16 hours.

### GST Pull-down assays

GST fusion proteins were expressed and purified as per [30]. GST pull-down experiments were performed by mixing GST fusion proteins with cell lysates rocked at 4°C overnight, washed 3 times with PBS, and then boiled in SDS sample buffer. Associated proteins were resolved by SDS-PAGE, followed by immunoblotting. GST fusion protein purity after glutathione bead purification was measured by SDS-PAGE and Coomassie stain. Each GST fusion protein was seen as a single band, with no contaminating bands, and an equal amount of each fusion protein was used for pull down assays.

### Co-Immunoprecipitation and immunoblotting

Co-immunoprecipitation experiments were performed essentially as described in [30]. Briefly, protein from cultured cells was extracted in lysis buffer (20 mM Tris-Cl, pH 7.5, 0.137 M NaCl, 2 mM EDTA, 1% Triton, 10% glycerol, 25 mM beta glycerol phosphate, with 1 mM phenylmethylsulfonyl fluoride and 1x protease inhibitor mixture [30]), and the lysates were incubated overnight at 4°C with 5 μg of nonimmune rabbit IgG, or goat anti-striatin antibody (Santa Cruz). Protein G beads (Amersham Biosciences) were then added and a further incubation carried out at 4°C for 2 h. The pellets obtained after centrifugation were washed five times with wash buffer (50 mM Tris, pH 7.5, 7 mM MgCl_2_, 2 mM EDTA, and 1 mM phenylmethylsulfonyl fluoride). The washed immunopellets were resuspended in SDS-PAGE sample buffer, and proteins resolved by SDS-PAGE, transferred to nitrocellulose membranes, and then probed with the appropriate primary antibody. Antibodies used are: rabbit polyclonal anti-ERα HC20 (Santa Cruz Biotechnology), anti-striatin (BD Transduction Laboratories), and rabbit polyclonal anti-phospho-AKT, phospho-ERK, phospho-eNOS, total AKT, total ERK and total eNOS (Cell Signaling Technology), and mouse monoclonal anti-GAPDH (Calbiochem). The membranes were then incubated with the appropriate HRP-linked secondary antibody (GE Healthcare) and developed with ECL (Amersham Biosciences).

### Transient transfections & luciferase reporter assays

To test for genomic functions of WT and KRR mutant ERα, stable Eahy926 cell lines (Ctrl_hEC, WT_hEC and KRR_hEC) were grown in phenol red-free DMEM with 10% charcoal-stripped bovine growth serum (sBGS) for 24 hours, and transiently transfected with an ERE-luciferase reporter plasmid and β-galactosidase expression plasmid [27] using PolyFect reagent (Qiagen). 6 hours after transfection, cells were switched to serum free medium containing 10 nM 17β-estradiol (E2, Sigma-Aldrich, St. Louis, MO) or ethanol vehicle for 16 hours, and normalized luciferase/β-gal values determined, as per [31].

### qRT-PCR

Total RNA was purified with the RNeasy mini kit (Qiagen). RNA was reverse transcribed by using of the QuantiTect reverse transcription kit (Qiagen) and qRT-PCR was carried out using SYBR Green (QIAGEN) and the following human gene-specific primers: HAVCR2 Fwd: TGCAATGCCATAGATCCAAC & Rev: GGCAATGACATGCCTGTTTA, PTGS2 Fwd: GTTGGAGCACCATTCTCCTT & Rev: GGACAGCCCTTCACGTTATT, RGS4 Fwd: GCGAATTCCAAGCTGTTAAA & Rev: TTGACTTCCTCTTGGCTCAC, VASN Fwd: ATGTGCTCCAGGGTCCCT & Rev: GAGCATGGTGATGCCGTT. Relative expression of target genes was calculated with the comparative CT method, with normalization to GAPDH (Fwd: GAGCCAAAAGGGTCATCATCTCT & Rev: GGGTCTCTCTCTTCCTCTTGTGC).

### Microarray analysis

RNA from three separate isolates of each Eahy926 stable cell line (WT_hEC and KRR_hEC) was processed and hybridized to Illumina HumanHT-12 v4 Expression BeadChip microarrays at the Yale Center for Genome Analysis (YCGA, http://medicine.yale.edu/keck/ycga/microarrays/index.aspx). Differential expression was determined using Limma [33]. Genes showing at least a 1.3 fold change in expression WT_hEC+E2—WT_hEC+Veh, or KRR_hEC+E2—KRR_hEC+Veh, and a p. value < 0.01 were considered to be significantly regulated by E2 in WT or KRR mutant ERα-expressing hECs, respectively. Raw and processed microarray data are available on GEO under accession number GSE72180. Predicted functions of E2 regulated genes in WT hECs were examined using QIAGEN’s Ingenuity® Pathway Analysis (IPA®, QIAGEN Redwood City, CA).

### EC migration and proliferation assays

To measure migration, Ctrl_, WT_, and KRR_hECs were grown to 80–90% confluence in a 6-well plate (BD Bioscience). After 24 hour incubation in serum free medium, a scratch “wound” was made with a plastic p200 tip, and the wells were rinsed 3 times to remove non-adhered cells, followed by treatment with 10 nM E2 or ethanol vehicle for 48 hours in 0.5% charcoal-stripped FBS medium. Live images were taken at 0 and 48 hours with a Nikon Ti-Eclipse microscope with a 10x objective lens connected to a Photometrics Coolsnap EZ Turbo 1394 camera. Cells that migrated into the wound area were counted at eight different locations along the scratch and averaged. To measure proliferation, Ctrl_, WT_, and KRR_hECs were seeded in a 96-well plate at a density of 2500 cells/well in standard growth medium. After plating, the media was replaced with phenol red free DMEM with 1% charcoal-stripped FBS and with either 10 nM E2 or vehicle. Cell proliferation was quantified using a Cell TiterGlo Luminescent Cell Viability assay (Promega) at day 3.

### Measurement of monocyte adhesion to ECs

Ctrl-, KRR- or WT-ECs were grown to 80% confluence in 10% charcoal stripped BGS, switched to serum free medium for 24 hrs, and then treated for 16 hours with 10 nM E2 or ethanol vehicle in serum free medium. Monocyte adhesion was measured essentially as described in [34]. Briefly, U937 monocytic cells were maintained in suspension culture in RPMI-1640 with 10% BGS, 2 mm L-glutamine and 10 mm HEPES, at 37°C. Exponentially growing U937 cells were harvested, washed twice with PBS and labeled with BCECF-AM (2μM) (Molecular Probes, Eugene, OR) in PBS for 30 min at 37°C. Equal numbers of fluorescently-labeled U937 cells were incubated with EAhy stable cells for 2h. Cells were gently washed three times with PBS, to remove non-adherent cells. Adherent fluorescent cells were counted for five fields, and counts were averaged for each biological replicate. The reader was blinded to sample treatments.

### Transcription factor binding site analysis

TFBS enrichment was determined using our established methods [35]. Briefly, the promoter DNA sequences of ERα rapid signaling up-regulated genes (regulated by E2 in WT hECs but not KRR hECs), from -1000 to +200 bp, were scanned for homology to TFBS matrices from Transfac and Jaspar/non-redundant databases with Storm [36], using a threshold p. value of 0.0005. The same analysis was also performed for over 800 control promoter sequences from genes that were not regulated by E2 (fold change <1.05 and p. >0.1). The significance of differences between foreground and background values for matrix matches was assessed using binomial tests, adjusted for multiple testing using the Benjamini-Hochberg method, and TFBS matrices showing fold-enrichment of >1.15 and adjusted p. < 0.05 were identified as significantly enriched. If more than one matrix existed for the same TF (e.g. V$MMEF2_Q6, V$MEF2_02, Jaspar$MEF2A, etc.) we examined the enrichment for all such matrices (even those that did not meet our initial significance requirements). In order to avoid biasing the results towards a possible small subset of outlier matrices for a given TF, we required that all matrices for a TF show at least a 1.05-fold enrichment, in order for any matrix for that TF to be called as significantly enriched. The relatedness of all significantly-enriched matrices was determined using STAMP [37].

### Statistical analyses

Data are represented as the mean +/- the standard error of the mean (SEM). The significance of differences was assessed using t-tests adjusted for multiple testing using the Benjamini-Hochberg method, with an adjusted p. value of <0.05 considered to be significant. Each experiment was performed a minimum of three independent times. The statistical approaches used for the microarray and TFBC analyses are described in those sections, above.

## Results

### Identification of KRR mutation in ERα that blocks its interaction with striatin

**Fig 1:**
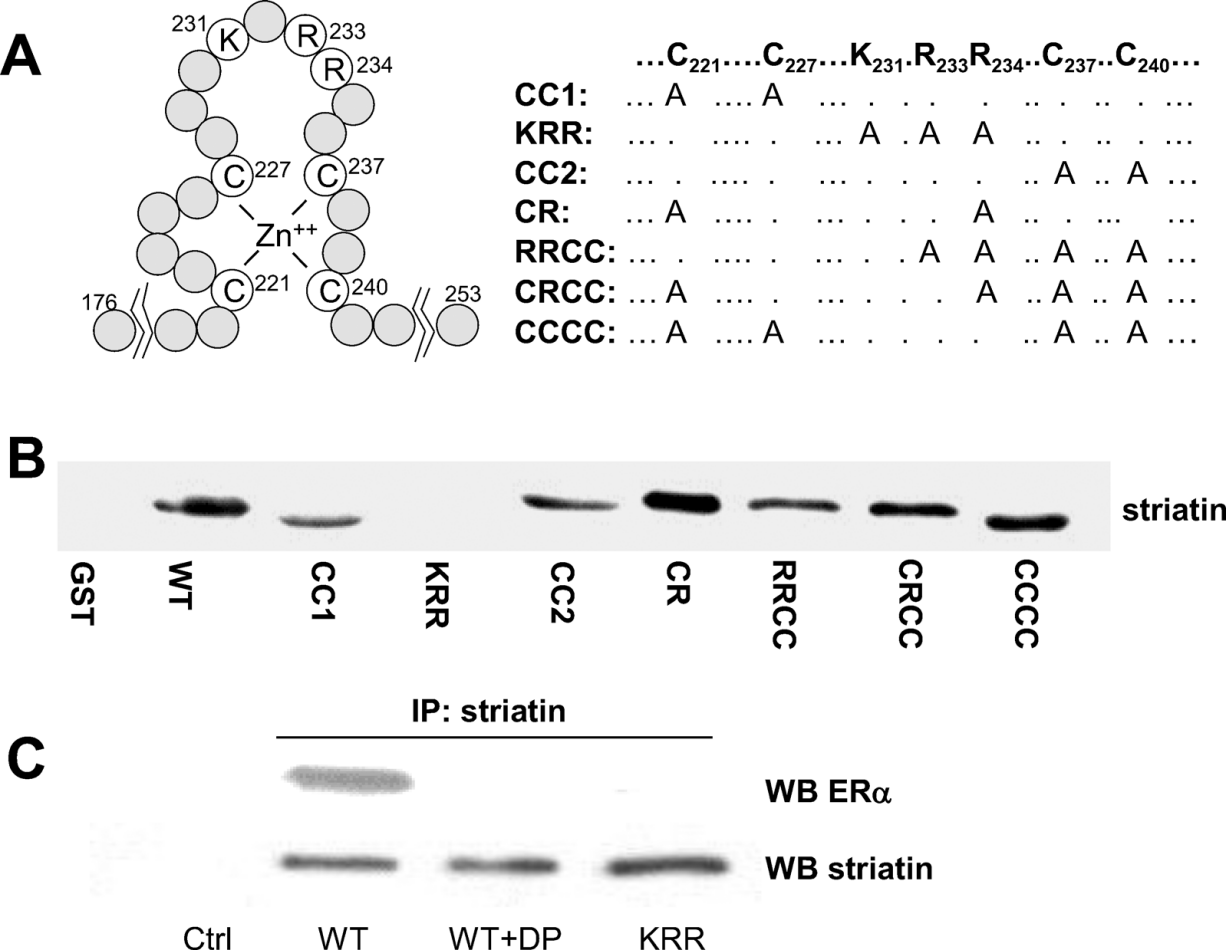
Identification of striatin non-interacting KRR mutant of ERα. **(A)** Locations of mutations to alanine in the ERα 176–253 disrupting peptide. **(B)** GST pull down assay showing the interaction between GST fusions to each WT or mutant ERα peptide with His-tagged striatin AAs 1–203. **(C)** COS-1 cells were co-transfected with plasmids expressing WT ERα, WT ERα & the disrupting peptide (DP) or KRR mutant ERα. A co-immunoprecipitation experiment was then performed, where extracts were immunoprecipitated with anti-striatin antibody and then Western blotted with the indicated antibodies. Ctrl: control IP from a mixture of all 3 extracts with non-immune IgG.

The disrupting peptide used to block ERα:striatin interactions in the DPM model contains the amino acids 176–253 of ERα [20, 27]. We performed a mutagenesis scan of this peptide to identify specific amino acids required for ERα:striatin interactions (as summarized in Fig 1A). We found that one particular mutation, a triple point mutation of ERα amino acids lysine 231, arginine 233 and arginine 234 to alanine (KRR->AAA), alone amongst the mutations tested, essentially eliminated interactions between the peptide and striatin (Fig 1B & DNS). When expressed in COS-1 cells full-length WT ERα interacted with endogenous striatin (as measured by co-IP), but full length ERα containing the KRR mutation did not, similar to what is seen when WT ERα is co-expressed with the 176–253 disrupting peptide (Fig 1C).

**Fig 2:**
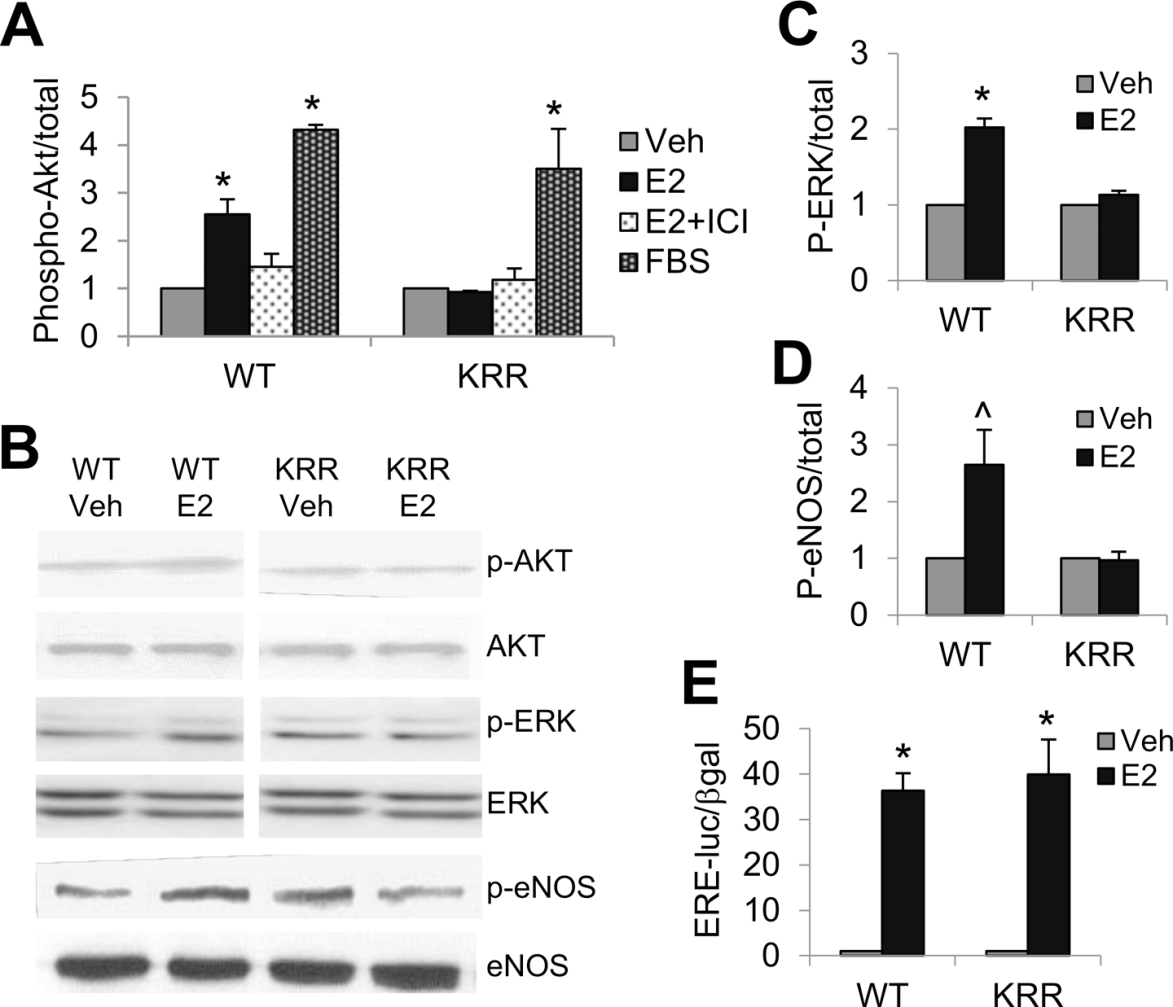
Transiently expressed KRR mutant ERα loses rapid signaling but maintains genomic signaling, in COS-1 cells. **(A)** Quantification of Western blots for phospho-Akt relative to total Akt in COS1 cells transiently transfected with WT or KRR mutant ERα expression plasmids, and then treated for 20 minutes, as indicated, with 10 nM E2, with or without 1 μM ICI 182780 (TOCRIS), ethanol vehicle, or 10%FBS. **(B)** Representative blots for the data summarized in (A), (C) & (D). **(C)** As in (A), but measuring phospho-ERK versus total ERK. **(D)** Phospho-eNOS relative to total eNOS in COS1 cells transiently transfected with an eNOS expression vector and either the WT or KRR mutant ERα expression vector. **(E)** COS-1 cells were transiently co-transfected with an ERE-driven luciferase reporter plasmid and β-galactosidase expression plasmid, and with either WT ERα or KRR ERα expression plasmids. β-gal-normalized luciferase activity was measured after 16hr treatment +10nM E2 or +Veh. Results were normalized to the +Veh condition. *; p<0.05, ^; p = 0.06. Bars; standard error.

COS-1 cells transiently transfected with a plasmid expressing WT ERα showed rapid signaling responses to E2, indicated by the ability of a 20-minute E2 treatment to promote phosphorylation of Akt, ERK and eNOS, but this response was absent in cells transfected with KRR mutant ERα (Fig 2A–2D). Activation of Akt by E2 in WT ECs was estrogen-receptor specific, since it was blocked by the specific ER antagonist ICI182780. In addition, the lack of Akt activation by E2 in KRR hECs was not due to a defect in kinases upstream of Akt, since growth factors in serum (FBS) could activate Akt in both WT and KRR hECs (Fig 2A). By contrast, both WT and KRR mutant ERα supported genomic signaling through binding to estrogen response elements (EREs) in DNA, as measured by the ability of 16 hour E2 treatment to induce luciferase expression from a transiently transfected ERE-luciferase plasmid in COS-1 cells (Fig 2E).

### Stable EC lines bearing KRR mutant ERα maintain genomic signaling through EREs but lose rapid signaling

**Fig 3:**
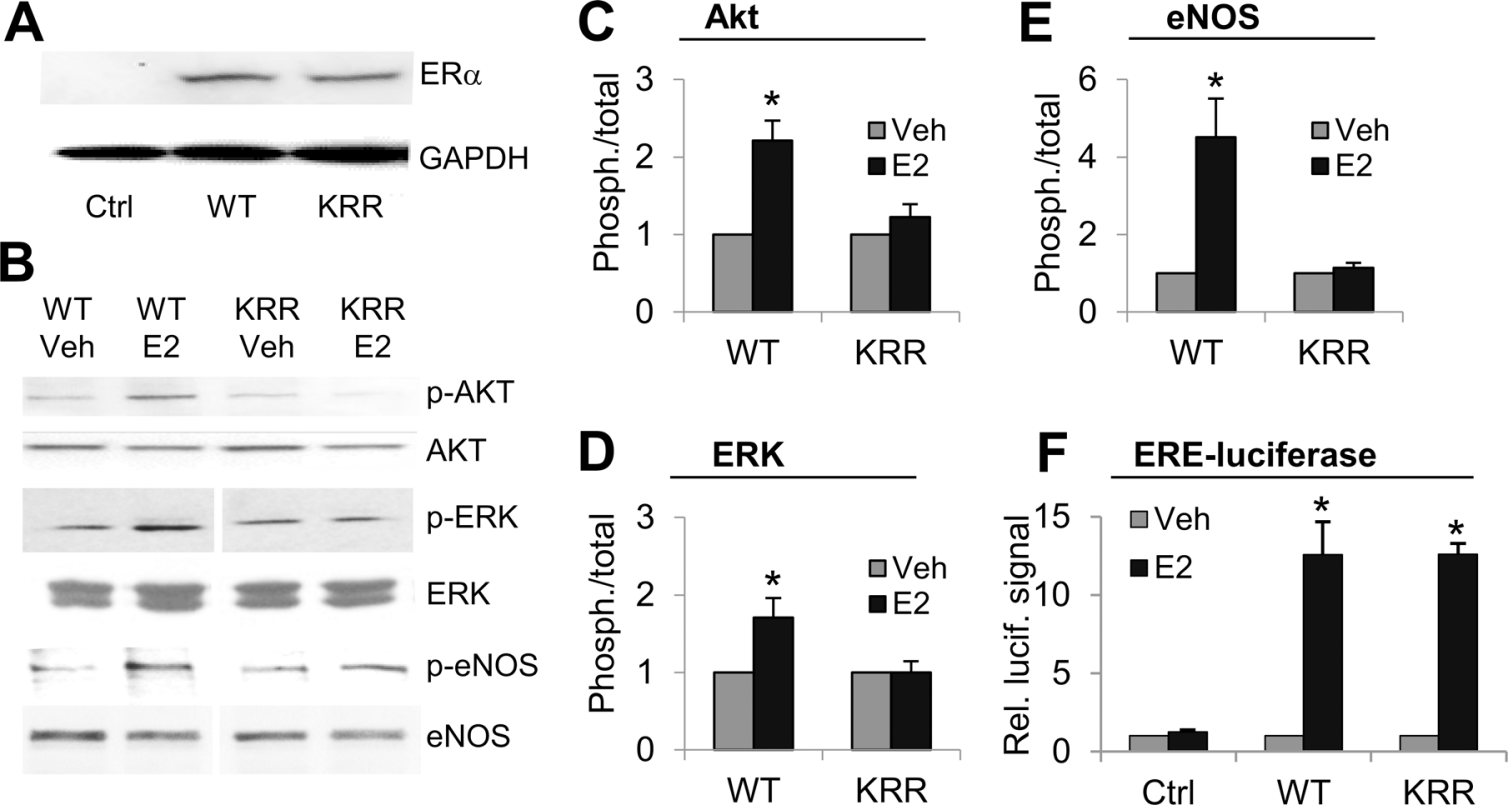
KRR mutant ERα in stable EC cell lines activates genomic but not rapid signaling. **(A)** Western blot for ERα or GAPDH (as an internal control) in Ctrl, WT or KRR stable Eahy926 cell lines. All WT or KRR clones had similar levels of ERα expression, while all Ctrl hEC clones had no detectable ERα expression (DNS). **(B-E)** WT or KRR hECs were treated +E2 or +Veh for 20 mins, followed by Western blotting for total versus phosphorylated active forms of endogenous Akt **(C)**, ERK **(D)** or eNOS **(E)**, as per Fig 2A–2D, with representative images in **(B)**. **(F)** WT or KRR hECs were transfected with ERE-luc & β-gal reporter plasmids, as per Fig 2E. *: p. <0.05. All WT or KRR clones had similar levels of ERE-binding genomic ERα function, measured as E2-induced luciferase expression in this assay, while all Ctrl hEC clones had no significant E2-induced luciferase activity (DNS).

Estrogen promotes re-endothelialization after vascular injury, in vivo [12], and this effect could contribute to its ability to suppress smooth muscle cell proliferation and longer term, adverse vascular remodeling [6, 10, 11]. To create a model system for testing the specific function of rapid signaling through ERα in vascular endothelial cells, we created three stable derivatives of the Eahy926 immortalized human EC cell line, containing either an integrated empty expression vector (Ctrl_hECs), a human, full-length, wild type ERα expression vector (WT_hECs), or a full length KRR mutant ERα expression vector (KRR_hECs). While Ctrl_hECs have no detectable ERα expression, WT_hECs and KRR_hECs expressed similar levels of full length human ERα from stably-integrated transgene vectors (Fig 3A). Note that, parent Eahy926 cells and Ctrl_hECs have no detectable ERα or ERβ expression (Fig 3A, and data not shown). We found that WT ERα in WT_hECs activates rapid signaling to Akt, ERK and eNOS in response to E2, but that KRR mutant ERα lacks all of these rapid responses (Fig 3B–3E). On the other hand, both WT and the KRR mutant ERα are functional for genomic signaling through binding to ERE elements, as indicated by the ability of both WT_hECs and KRR_hECs, but not Ctrl_hECs, to activate transcription from an ERE-luciferase reporter plasmid (Fig 3F).

### Comparison of ECs bearing WT vs. KRR ERα indicates requirement of rapid signaling for majority of gene regulation by E2/ERα

**Fig 4:**
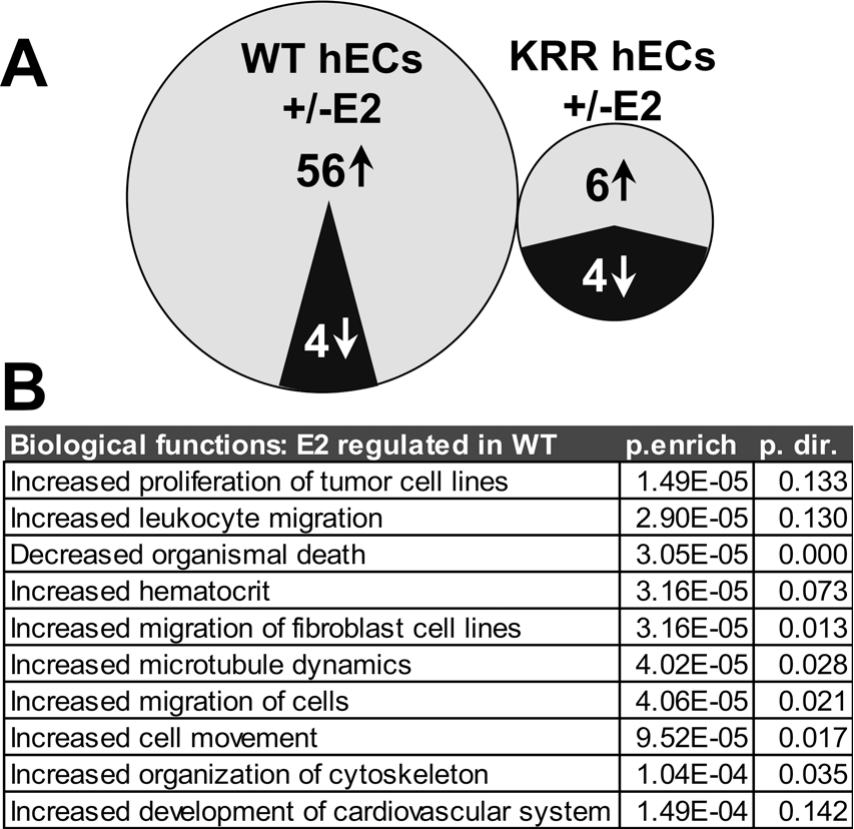
Most E2/ERα -regulated genes in hECs require rapid signaling, and rapid signaling-dependent genes are associated with increased cell migration and proliferation. **(A)** Venn diagrams depicting the number of genes upregulated or downregulated by 16 hr treatment with 10nM E2 in WT_hECs or in KRR_hECs, and the lack of overlap between these gene sets. **(B)** Biological functions associated with genes regulated by E2 in WT but not KRR hECs. “p. enrich” and “p. dir” are the p. values for enrichment of WT hEC E2-regulated genes with genes associated with that function, and for the predicted direction of effect, respectively.

To test the importance of rapid signaling in the transcriptional response of ECs to E2, we performed gene expression microarray analysis on WT and KRR hECs treated for 16 hrs +/- 10 nM E2. We found that 60 genes were regulated by E2 treatment in WT_hECs (56 upregulated and 4 downregulated, p. < .01 and fold change > 1.3). By contrast, only 10 genes were regulated by E2 in KRR_hECs (6 up and 4 down, Fig 4A). Complete lists of genes significantly regulated by E2 in WT ECs or in KRR ECs are provided in S1 Table. qRT-PCR confirmation of these results for four of four genes tested that were regulated by E2 in WT but not Ctrl or KRR hECs (three upregulated and one downregulated) is shown in S1 Fig. Interestingly, none of the genes that were regulated by E2 in WT hECs were also regulated by E2 in KRR hECs, suggesting that most of the normal gene regulatory effects of E2-bound ERα require rapid signaling. In addition, genes affected by E2 in WT hECs are predominantly upregulated, while those in KRR hECs are roughly equally up- and down-regulated, suggesting that rapid signaling primarily contributes to gene activation by E2. To explore the possible biological functions of gene regulation by E2/ERα rapid signaling in ECs, we used Ingenuity Pathway Analysis to identify “functions annotations” (groups of genes associated with particular biological functions) that were significantly associated with genes regulated by E2 in WT but not in KRR hECs. This analysis predicted that rapid signaling, specifically through E2 binding to ERα in ECs, will promote cell migration and proliferation (Fig 4B).

### Genes regulated by rapid signaling through ERα show significant enrichment of transcription factor consensus sequences

**Fig 5:**
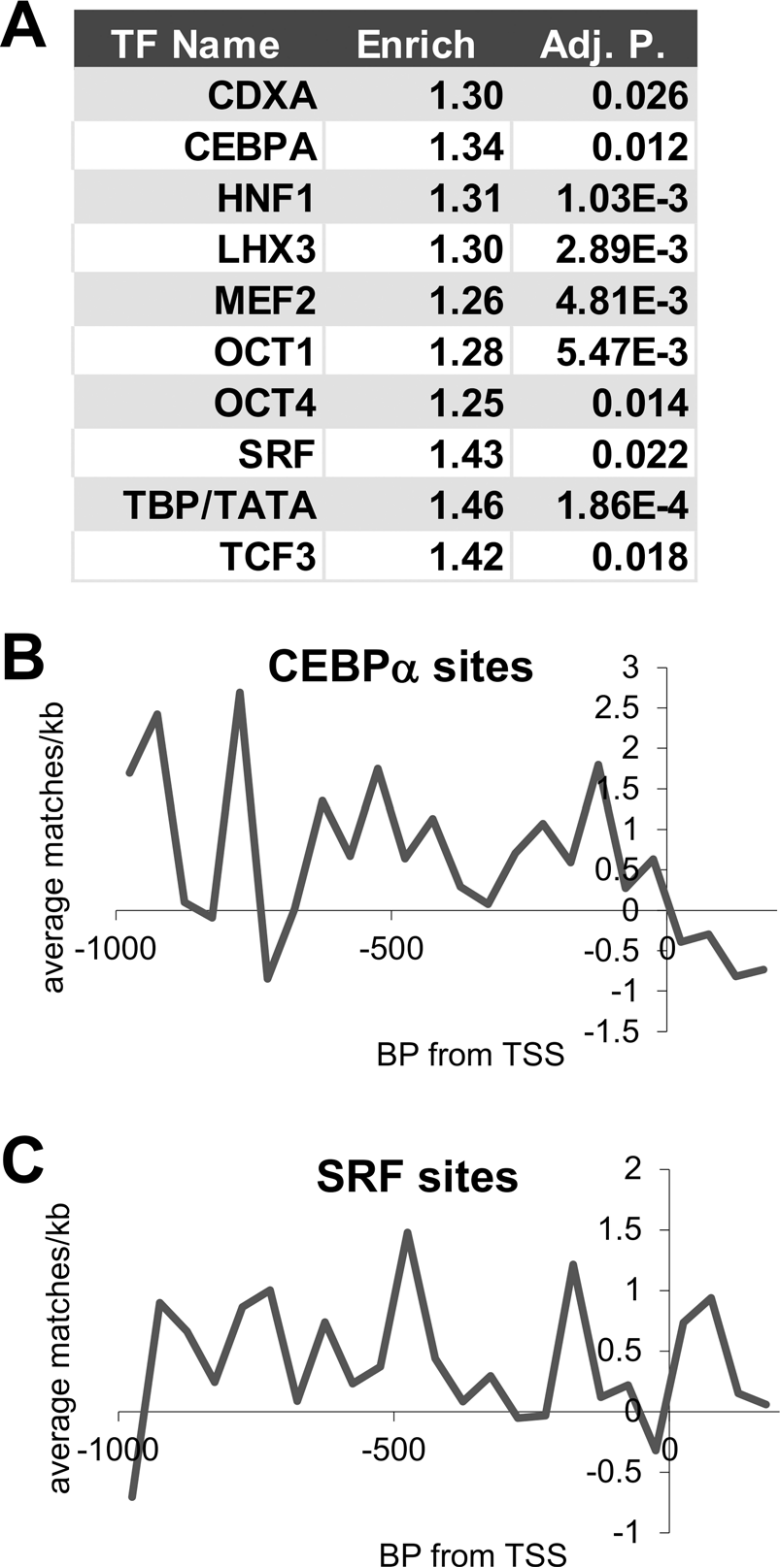
Distinct TF binding site enrichments in hEC genes regulated by E2/ERα rapid signaling. **(A)** Fold enrichments, and median adjusted p. values for every TF represented by more than one significantly-enriched TFBS matrix (adjusted p. value < 0.05 and enrichment over background, non-regulated gene promoters > 1.15), and which showed at least a 1.05-fold enrichment for all its cognate matrices. **(B & C)** Plots of the average frequency of matches to the significantly enriched CEBP and SRF matrices versus the TSSes of WT hEC E2 up-regulated genes, relative to the background from unregulated gene promoters.

We applied bioinformatic approaches to identify transcription factors (TFs) that are likely to mediate gene regulation by E2/ERα rapid signaling in ECs. While there were too few downregulated genes to perform this analysis, we found that the promoters of genes that were upregulated by E2/ERα rapid signaling (up-regulated by E2 in WT but not KRR hECs) show enrichment for distinct sets of TFBCs (Fig 5A), suggesting TFs that could act downstream of membrane bound E2/ERα to regulate rapid-signaling dependent target genes. The average enrichment profile across all upregulated promoters for CEBP and SRF matrices are shown in Fig 5B and 5C. A complete listing of all significantly enriched TFBC matrices is given in S2 Table. The relatedness of these matrices to each other is shown in S2 Fig.

### KRR ECs lack E2-stimulated proliferation and migration responses

**Fig 6:**
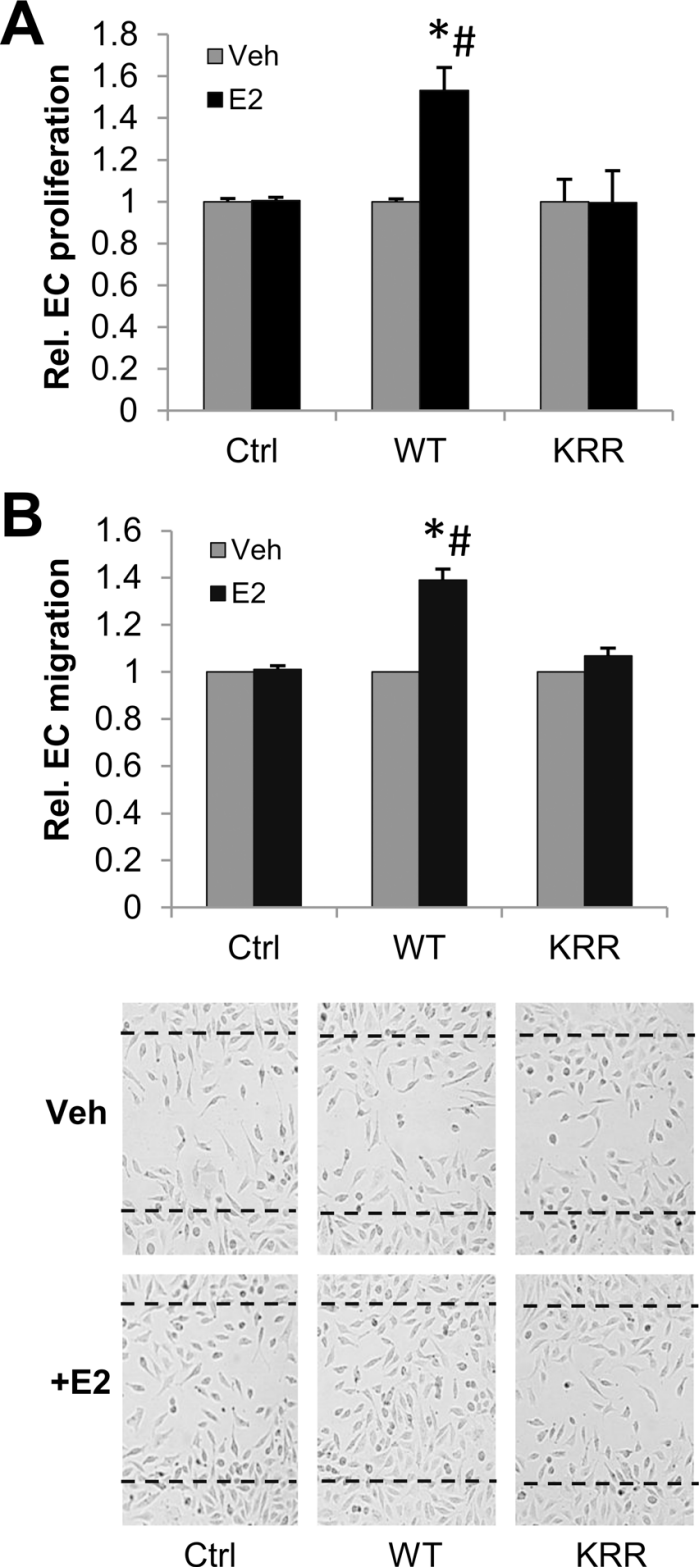
Rapid signaling through ERα is required for E2-dependent EC proliferation & migration. **(A)** Relative cell counts for stable Ctrl, WT or KRR hEC cell lines +/- 10nM E2, normalized to counts +Veh. **(B)** A “scratch” wound was made with a pipette tip on Control, WT or KRR hECs at near confluence, and the number of cells migrating into the scratch after 48 hours counted. Top: Quantitation of migration data. Bottom: representative images of cells after 48 hr migration. Dotted lines indicate the borders of the scratch at time 0. Data is normalized to +Veh for each cell line. *: different from WT+Veh, p. <0.05, #: different from KRR+E2, p. <0.05.

Using the stable Ctrl_hECs, WT_hECs and KRR_hECs as a model, we tested whether the rapid signaling functions of ERα were required for the ability of E2 to promote EC proliferation. We found that E2 increased proliferation of WT hECs, but that this response was lost in KRR hECs, similar to Ctrl_hECs lacking ERα expression (Fig 6A). Next, to test whether the promotion of EC migration by E2 requires rapid signaling through ERα, we measured the ability of Ctrl, WT or KRR hECs to migrate into a “scratch wound” on the culture dish. While E2 increased the migration of WT hECs, this response was lost in KRR hECs, again similar to Ctrl hECs lacking ERα expression (Fig 6B).

### E2 decreases monocyte adhesion to WT but not KRR ECs

**Fig 7:**
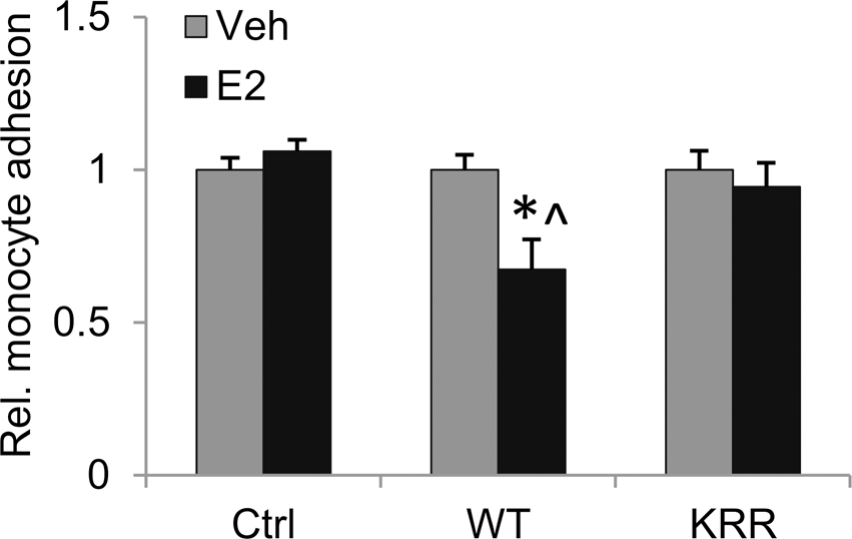
Rapid signaling through ERα is required for E2-dependent inhibition of monocyte adhesion to ECs. Relative counts of adherent U937 human monocytes to WT or KRR stable hECs +/- 10 nM E2, normalized to counts +Veh. *: different from WT+Veh, p. <0.05, ^: p. 0.06 versus KRR+E2.

Prior studies have shown that mechanical injury to the carotid artery in rats and mice induces the production of inflammatory cytokines and adhesion molecules (mRNA and protein), and infiltration of monocytes, neutrophils and T cells at times ranging from 1 hour to several weeks after injury [15, 16, 38–41]. Several of these effects were shown to be reduced by pre-treatment with E2 in a rat carotid injury model [15, 16], indicating that estrogen protects against injury-induced arterial inflammation. Furthermore, studies using knockout mice and other approaches indicate that inflammatory cytokines and chemokines, and their receptors, promote pathological remodeling after vascular injury and atherosclerosis [42]. To examine whether rapid signaling through ERα might play a role in anti-inflammatory responses of endothelial cells to E2, we assayed for the ability of human monocytes to adhere to Ctrl, WT or KRR hECs. We found that E2 significantly decreased monocyte adhesion to WT hECs, relative to Veh treated WT or to Ctrl hECs (Fig 7). Notably, this response to E2 was lost in KRR hECs, suggesting a role for rapid signaling through ERα in mediating the anti-inflammatory effects of E2.

## Discussion

The effects of estrogen in endothelial cells could be mediated by ER binding to DNA (the classic genomic pathway) or by rapid signaling through ER to activate cellular kinases. It has been widely assumed that the gene regulatory and lasting physiological effects of estrogen would be controlled by genomic signaling, with no significant contributions from rapid signaling. To examine the specific roles of rapid signaling in ECs, we have identified a KRR mutant form of ERα that is specifically deficient in rapid signaling (but not genomic signaling through EREs), and characterized EC lines expressing WT versus this KRR mutant ERα. Our results indicate that rapid signaling is essential for gene regulation by E2/ERα in ECs, is required for E2 dependent promotion of EC proliferation and migration, and is required for E2 dependent inhibition of monocyte adhesion to ECs. These observations indicate that, contrary to the dominant paradigm, rapid signaling through ERα will be essential for many of the important effects of E2 on ECs, including promotion of re-endothelialization and inhibition of vascular inflammation.

These observations are consistent with, and expand on, our recent studies, which used a disrupting peptide to block rapid signaling by inhibiting the interaction between ER and the critical adaptor molecule striatin [20]. Those studies demonstrated that disruption of rapid signaling greatly altered gene regulation by E2 in mouse aorta, eliminated the ability of E2 to stimulate EC migration and proliferation, and removed the vasculoprotective effects of E2 in an arterial wire injury model. However, the blocking peptide in those studies inhibited the interaction of both ERα and ERβ with striatin [20, 30], inhibited the binding of PP2A to the same domain of striatin [19], and could potentially block other protein’s interactions with striatin. Accordingly, those studies could not positively identify the functions of rapid signaling through ERα. By contrast, KRR mutant ERα loses the ability to bind striatin but does not affect the interaction of striatin with other proteins, providing a much more precise tool to identify the specific rapid signaling functions of ERα. Note that the ERE-luciferase data in Figs 2 and 3 indicate that genomic signaling mediated by ERα binding directly to EREs, together with gene activation via interaction with appropriate co-activators, is intact in KRR mutant ERα. However, we cannot rule out the possibility that the KRR mutation could subtly alter affinities for different types of EREs (e.g. EREs with different spacing between half sites). Furthermore, genomic functions of ERα can also be mediated by other mechanisms, such as by tethering to other DNA bound TFs, and we cannot rule out the possibility that the KRR mutation might affect a subset of these genomic signaling functions.

Whereas E2 treatment of ECs containing WT ERα promotes their migration and proliferation, KRR mutant ERα was essentially equivalent to the lack of ERα, showing no E2 effect in these assays. This suggests that rapid signaling through ERα is specifically required for these endothelial cell responses to E2. In addition, prior studies have shown that E2 can attenuate lipopolysaccharide (LPS) induced monocyte adhesion to cultured ECs [43]. Here we demonstrate that monocytes adhere to ECs expressing WT ERα even under basal, non-LPS stimulated conditions, and that this is inhibited by E2. By contrast, both Ctrl_hECs and KRR_hECs lose this E2 response. This indicates that inhibition of monocyte adhesion to vascular endothelia by E2 may require rapid signaling through ERα, and suggests that the ability of E2 to decrease vascular inflammation after injury, in vivo, may also require rapid signaling [16, 44]. Furthermore, inflammation early after injury is likely to play a role in long term vascular remodeling responses after injury. Thus, a potential role for rapid signaling through ERα in suppressing inflammation could contribute to the requirement, observed in our recent disrupting peptide mouse studies, for rapid signaling through ERα or ERβ in E2 dependent suppression of SMC proliferation and vascular remodeling two weeks after injury [20].

We found that the number of genes regulated by E2 in KRR_hECs was six times less than that in WT_hECs, and that none of the genes that were regulated by E2 in WT_hECs were regulated by E2 in KRR_hECs. This suggests the response of most E2/ERα-regulated genes in ECs requires rapid signaling through ERα, without which they lose E2 responsiveness. Interestingly, the effect of ERα rapid signaling on gene regulation was predicted by IPA analysis to promote cell proliferation and migration. Indeed, genes that were upregulated by E2 in WT hECs but not in KRR hECs included: PDGFB, a growth factor that promotes proliferation and migration of ECs [45], PTGS2/Cyclooxygenase 2, whose product, prostaglandin E2 can auto-stimulate ECs to proliferate and migrate [46], and PFKB3/phosphofructokinase 3, an apparent glycolytic enzyme that was recently found to localize to the nucleus to promote cell proliferation [47]. Taken together with our observations that E2 can promote the migration and proliferation of WT but not KRR_hECs, these results suggest that gene regulation mediated by rapid signaling through E2-bound ERα functionally promotes EC proliferation and migration.

We found that EREs were not enriched in E2/ERα rapid signaling-upregulated promoters in ECs, arguing against gene regulation primarily through direct E2/ERα genomic effects. Instead, we found enrichment of binding site consensus sequences for several other TFs. Notably, some of these TFs are known to be regulated downstream of E2/ER rapid signaling pathways, including FOXO [48] and SRF [49, 50], and others are known to be phosphorylated and regulated by rapid signaling kinases, including CEBP [23, 51, 52], MEF2 [53–56] and TBP [51, 57]. These observations provide initial insights into the transcription factors that may convert rapid signaling effects from membrane bound ERα to long term gene regulatory responses in vascular cells.

Note that, while these studies indicate a requirement for rapid signaling through striatin for proper gene regulation by E2-bound ERα in ECs, they do not tell us which downstream effector kinase, or which phosphorylation targets, mediate these effects. It will be important, in future studies, to determine these downstream pathways, since this could potentially allow specific targeting of the most relevant pathways with existing or potential pharmacological inhibitors or activators. As a first step in this direction, we tested whether the PI3K/Akt pathway was required for rapid-signaling dependent activation of the ERα target gene, endothelial nitric oxide synthase (eNOS), by E2. Briefly, in WT hECs, we found that E2 induced eNOS expression both at the protein and mRNA level (S3A & S3B Fig), consistent with other reports [58, 59]. In our prior studies, we had shown that induction of eNOS by E2 required striatin, and was inhibited by the striatin-ER disrupting peptide [20]. E2 also had no effect on eNOS protein levels in KRR hECs, indicating that eNOS induction by E2 requires rapid signaling of ERα through striatin. Addition of the PI3K inhibitor, Ly294002, however blocked E2-dependent induction of eNOS mRNA and protein in WT cells, suggesting that signaling through striatin to the PI3K/Akt pathway is essential for the induction of eNOS by E2/ERα.

**Fig 8:**
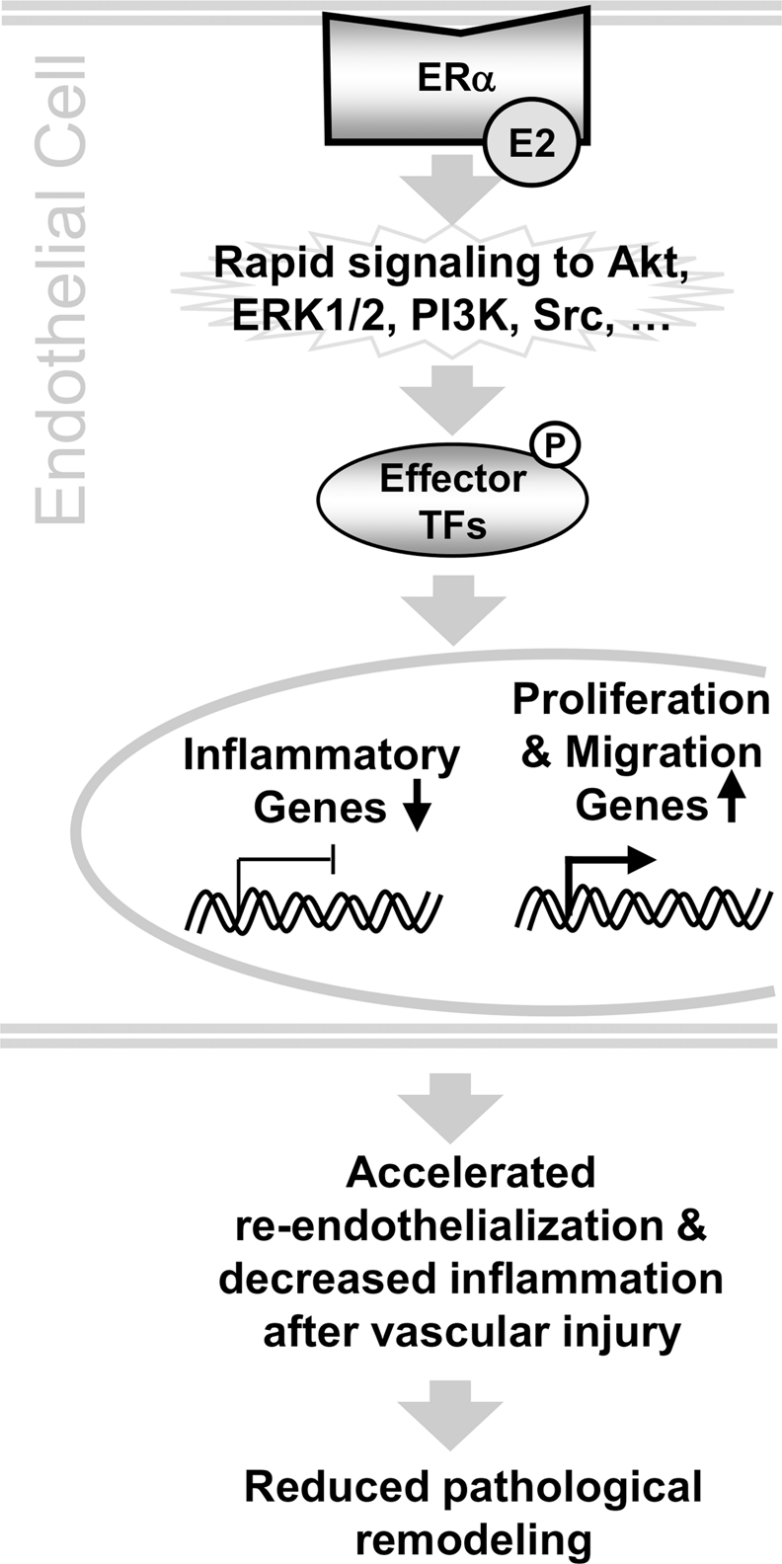
Model for gene regulation through E2/ERα rapid signaling. Rapid signaling from membrane bound E2/ERα, through striatin to Akt, ERK1/2, PI3K, Src and other cellular kinases, could phosphorylate and activate other TFs that regulate target genes that, then, promote EC proliferation and migration and reduce inflammatory responses in ECs. In vivo, these effects in ECs could promote re-endothelialization and inhibit long term pathological remodeling after vascular injury.

Overall these observations support a model in which E2 regulates transcription in ECs primarily through the rapid signaling pathway, in which E2 interaction with membrane bound ERα activates rapid signaling kinases to control the activities of other TFs (Fig 8). These TFs then alter the expression of target genes that promote EC proliferation and migration and inhibit inflammation, cellular effects that are expected to increase the rate of re-endothelialization and reduce inflammation after vascular injury. These positive responses in ECs early after vascular injury could then limit SMC proliferation and other long term adverse vascular remodeling responses to injury.

The Eahy926-derived stable cell lines we used here had no detectable expression of endogenous ERα or ERβ (Fig 3A & DNS). Furthermore, Ctrl-hECs showed no response to E2 in any assay (Figs 6 and 7, and DNS), indicating that the responses observed in WT-hECs were due to ERα expression and not due to endogenous expression of ERβ or of the seven transmembrane-domain estrogen receptor, GPR30. Furthermore, given that prior studies have shown that ERα is essential for the vasculoprotective effects of E2 [6, 10–12], we think that E2 signaling through ERα in ECs, examined here, is likely to be the most relevant mechanism of E2 action with regard to vascular disease and injury.

Our observations should be considered in the context of two recent, independent studies which generated transgenic mice in which the palmitoylation site on ERα was mutated (C541A), resulting in a decrease in ERα association with the plasma membrane and a loss of rapid signaling to AMPK and eNOS [29] or to Akt and ERK [60]. Neither report examined EC proliferation or monocyte adhesion. However, consistent with our observations in Eahy926 clones, Adlanmerini et al. found that primary aortic ECs from C541A animals no longer migrated in response to E2, and also that the ability of E2 to stimulate re-endothelialization after carotid artery injury was lost in C451A animals [29]. Interestingly, they found that uterine growth and morphology was normal in C451 mice, and, using expression arrays, that gene regulation by E2 in the uterus, in vivo, was generally not altered by the C451 mutation. This suggests that the importance of rapid signaling through ER varies by tissue or cell type: being essential for proper gene regulation in ECs (this report) and in whole aortas [20], but being largely dispensable for proper gene regulation in the uterus, where classical genomic pathways may predominate.

Our prior studies using the DPM mouse, showed that rapid signaling through ER (either ERα or ERβ) was *required* for E2 dependent inhibition of remodeling after injury and for EC proliferation and migration. Furthermore, the C451 studies indicate that rapid signaling is required for re-endothelialization after vascular injury [29]. While the DPM and C451 models specifically abrogate rapid signaling, other recent studies have used the complementary approach of selectively activating only ER rapid signaling, using estrogen dendrimer conjugates (EDCs), which cannot enter cells to stimulate genomic signaling. These studies showed that activation of only rapid signaling could regulate gene expression in cultured breast cancer cells [61], and could increase the rate of re-endothelialization after vascular injury and reduce the development of atherosclerosis, similar to the effects of E2 [62]. By contrast, selective activation of rapid signaling did not increase uterine mass or promote the proliferation of estrogen dependent breast tumors. Taken together, these prior studies indicate that rapid signaling through either ERα or ERβ is both *required for* and *sufficient for* many of the vasculoprotective effects of estrogen.

In summary, we find that E2/ERα rapid signaling is required for the gene regulatory and physiological responses of vascular ECs to estrogen. Considered in the context of other recent studies, these observations suggest that the selective dependence of vasculoprotection by estrogen on rapid signaling may allow the beneficial vascular effects of estrogen to be activated in the absence of associated negative effects in other tissues, and indicate that a better understanding of the mechanisms and functions of rapid signaling through ER has the potential to identify novel approaches for the prevention and treatment of vascular disease. The studies presented here move us towards this better understanding by beginning to identify mechanisms by which E2/ERα rapid signaling could regulate transcription to promote vasculoprotective responses of ECs to E2. They also identify and establish a novel, triple point mutant ERα, the application of which is expected to greatly facilitate the identification of ERα-specific vascular and non-vascular functions of rapid signaling, and the mechanisms underlying these functions.

## Supporting Information

S1 Fig qRT-PCR validation of microarray results. Four or more RNA samples (independent of those used for microarray library construction) were analyzed by qRT-PCR with primers to the indicated genes. Signal was normalized to GAPDH as an internal control and the E2/Vehicle ratio calculated. For each gene, the E2/Veh ratio in WT cells was significantly different from 1, and in the same direction as seen on the microarray. For comparison, the E2/Veh ratios from the microarray data were; PTGS2/COX2: 1.71, RGS4: 0.758, VASN: 2.59, and HAVCR2: 1.74. There were no significant differences between +E2 and +Veh conditions for Ctrl or KRR hECs. *: differs from +Veh, p. < .05, #: differs from KRR+E2, p. < .05. Bars: SEM.
(PDF)

S2 Fig Homology tree for all TFBC matrices that were significantly enriched in WT_hEC E2 versus vehicle treated cells. The sequence similarity of all of the significantly regulated TFBS matrices (from S2 Table) was examined using STAMP. The scale bar represents an 0.2 base difference in the weighted matrix consensus sequence.
(PDF)

S3 Fig Signaling through PI3K/Akt is required for the induction of eNOS by E2/ERα. WT or KRR hECs were treated with vehicle, 10 nM E2, or 10 nM and 30 μM Ly294002 (a PI3K inhibitor), in serum free medium, for 40 hours prior to harvest of protein for western blot with anti-eNOS antibody (BD Biosciences) **(A)**, or for 16 hours prior to harvest of RNA for qRT-PCR with the eNOS-specific primers F: ACCCTCACCGCTACAACATC, R: GCTCATTCTCCAGGTGCTTC **(B)**. *; different from +Veh, p. < .05.
(PDF)

S1 Table Gene regulation by E2/ERα in ECs requires rapid signaling. **(A)** Genes significantly regulated by E2 in WT hECs. **(B)** Genes significantly regulated by E2 in KRR hECs. Note that, for (A), seven genes were recognized by two different probes, and the probe that gave the lowest p. value for differential expression was reported. In each such case, fold change values for the two probes were always within 10% of each other. “Log2FC”: base 2 logarithm of the fold change in expression E2 versus vehicle. “Log2AvExp”: base 2 logarithm of the average normalized expression level (from microarray signal intensity) for both E2 and vehicle conditions.
(XLSX)

S2 Table TFBC matrices enriched in rapid signaling upregulated gene promoters. Each matrix that was significantly enriched in promoters that were regulated by E2 in WT hECs but not in KRR hECs (Benjamini Hochberg adjusted p. value < 0.05 and fold enrichment > 1.15) is given, but only if all matrices for the same transcription factor showed at least a 1.05-fold enrichment.
(XLSX)
