# Supplementary figures and images for: ER Alpha Rapid Signaling Is Required for Estrogen Induced Proliferation and Migration of Vascular Endothelial Cells
Source: PLoS One. 2016 Apr 1;11(4):e0152807. doi: 10.1371/journal.pone.0152807 (PMC4818104; doi:10.1371/journal.pone.0152807)

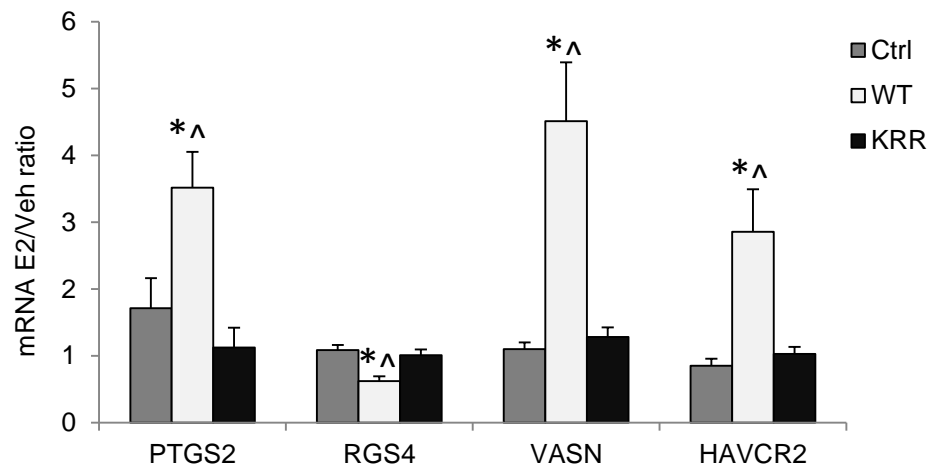

Supplement: S1 Fig — Four or more RNA samples (independent of those used for microarray library construction) were analyzed by qRT-PCR with primers to the indicated genes. Signal was normalized to GAPDH as an internal control and the E2/Vehicle ratio calculated. For each gene, the E2/Veh ratio in WT cells was significantly different from 1, and in the same direction as seen on the microarray. For comparison, the E2/Veh ratios from the microarray data were; PTGS2/COX2: 1.71, RGS4: 0.758, VASN: 2.59, and HAVCR2: 1.74. There were no significant differences between +E2 and +Veh conditions for Ctrl or KRR hECs. *: differs from +Veh, p. < .05, #: differs from KRR+E2, p. < .05. Bars: SEM. (PDF) [file pone.0152807.s001.pdf]

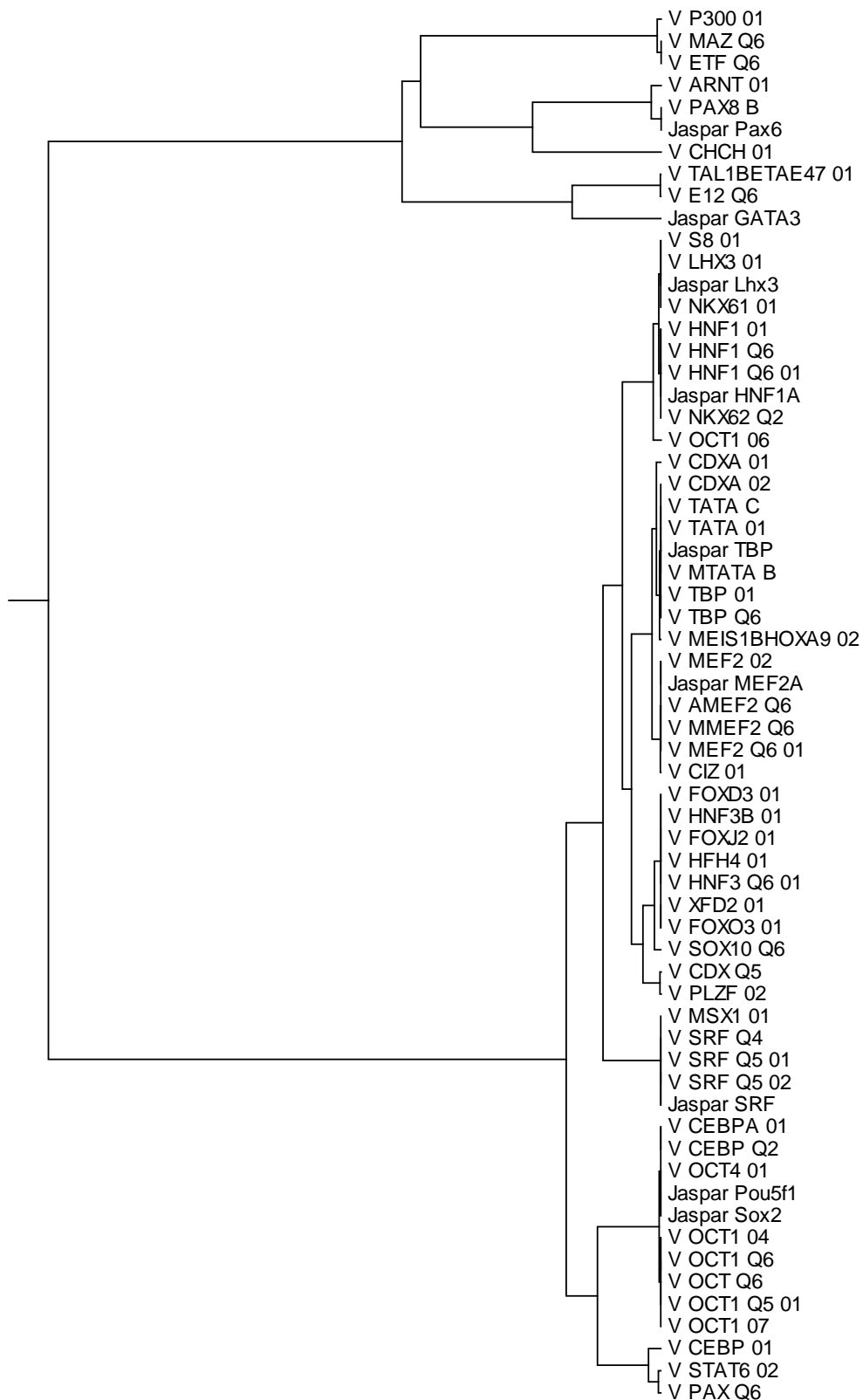

0.2

**Supporting Figure S2**

Supplement: S2 Fig — The sequence similarity of all of the significantly regulated TFBS matrices (from S2 Table) was examined using STAMP. The scale bar represents an 0.2 base difference in the weighted matrix consensus sequence. (PDF) [file pone.0152807.s002.pdf]

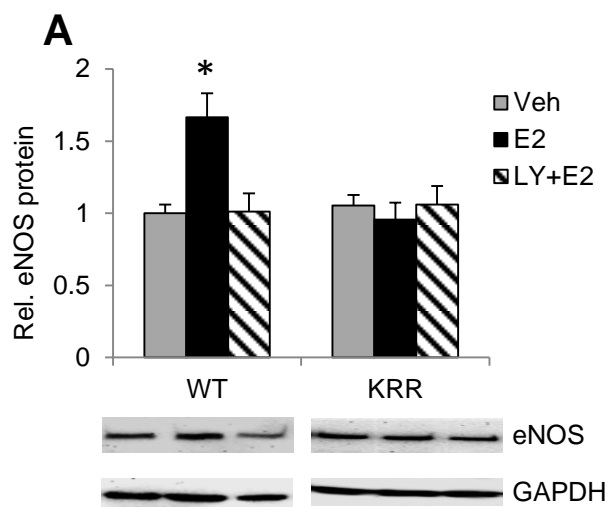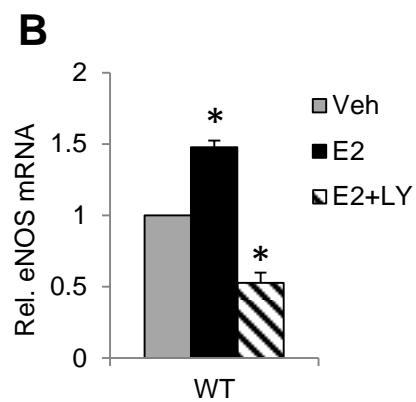

Supplement: S3 Fig — WT or KRR hECs were treated with vehicle, 10 nM E2, or 10 nM and 30 μM Ly294002 (a PI3K inhibitor), in serum free medium, for 40 hours prior to harvest of protein for western blot with anti-eNOS antibody (BD Biosciences) (A), or for 16 hours prior to harvest of RNA for qRT-PCR with the eNOS-specific primers F: ACCCTCACCGCTACAACATC, R: GCTCATTCTCCAGGTGCTTC (B). *; different from +Veh, p. < .05. (PDF) [file pone.0152807.s003.pdf]
